# Supplementary material for: Progress in survival in renal cell carcinoma through 50 years evaluated in Finland and Sweden
Source: PLoS One. 2021 Jun 22;16(6):e0253236. doi: 10.1371/journal.pone.0253236 (PMC8219161; doi:10.1371/journal.pone.0253236)
Supplement: Attachment — Submitted filename: 21rccREBUTTAL PONE.docx [file pone.0253236.s001.docx]

**REBUTTAL PONE-D-21-06223**

**Comments to the Author**

We thank the reviewers for clarifying comments. Our changes are highlighted in the manuscript.

As to a PONE general comment no. 2 we added statistical analysis details in Methods, bottom p. 3.

Reviewer #1: The authors used a database information to show the survival trend of kidney cancer in Finland and Sweden. It is an interesting observation study to see the improvement of survival in history. I have several comments as below.
1. Page 6, first paragraph, for treatment of mRCC, tyrosin kinase inhibitors are the major weapon since 2005.

>>> Addition on p. 4.

2. Please dress the limitations of this study since all the inference were according to past literature, not this data.

>>> Added to other limitations, p. 7

Reviewer #2: This study provide important information regarding survival of RCC.

minor comment:

1: please discuss in the era of target therapy ( like sunitinib , other TKI) and immunotherapy , how does this novel agents influence the survival of metastatic RCC in Finland and Sweden?
Did the NORDCAN database included these information?

>>> Addition on p. 3 and 6.

Reviewer #3: Hemminki et al., evaluated 50 years of survival data on RCC in Finland and Sweden.

In this descript analyses of data from two different countries both with free medical care show that in Finland there is an improvement in difference between 1- and 5- year survival data and such observation is missing in Sweden.

Comment: Authors documented reasonable explanations for the observed differences between Finland and Sweden and accounted for less healthcare spending in Finland during economic crisis. This economic crisis may be in the starting of 1990s. So comments need to be made whether the differences in survival after 1990s is significant between these two countries.

>>> Significance was described in the new paragraph, p. 5.

Reviewer #4: This manuscript presents valuable data which confirms the current knowledge regarding the natural history of renal cell carcinoma.

My comments are as follows:
The manuscript needs minor revision in terms of scientific writing.
The title talks about renal cell carcinoma but as far as I understood you have included Wilms tutor patients. I think one should be changed, either title or the analysis.

>>> 98.5% of cases are RCC, addition p. 3.
I honestly did not understand the purpose of section " Diagnostics and treatment for RCC". The fact that you mentioned this after methods part is a bit confusing.

>>> As this study covers 50 years we feel that it is important to highlight changes in diagnostic and treatment over this period which most readers do not know. We refer to this section also in Introduction, p. 3.

Reviewer #5: Studies on Renal Cell Carcinoma survival till date has shown favorable survival outcomes. However, these studies have been performed over limited periods of time. In this manuscript, authors examined survival rates in RCC over an extended period of time in Swedish and Finland populations. They analyzed RCC survival in Finland and Sweden (with similar health care systems) over a 50-year period (1967-2016) using data from the NORDCAN database provided by the local cancer registries.
In addition to the standard 1- and 5-year relative survival rates, they calculated the difference between 1 year and 5-year survival as a measure of how well survival was maintained between years 1 and 5. While relative 1-
year survival rates increased linearly in both countries and reached 90% in Sweden and 80% in
Finland, 5-year survival also developed favorably. The difference between 1- and 5-year survival rates did not improve in Sweden suggesting that the gains in 5-year survival were owing to gains in 1-year survival. In Finland there was a gain in survival between years 1 and 5, but the gain in 1-years survival was the main contributor to the favorable 5-year survival. The authors conclude that earlier diagnosis and surgical treatment of RCC is the main driver of the favorable change in survival during the past 50 years. This study reports novel findings. Though due to limited data available in NORDCAN database, there was lack of information on performed treatments and clinical stage, precluding the analyses of important potential variables.

>>> The lacking of individual data are acknowledged under limitations on p. 7.
